# The future of big data and artificial intelligence on dairy farms: A proposed dairy data ecosystem

**DOI:** 10.3168/jdsc.2025-0843

**Published:** 2025-10-25

**Authors:** Miel Hostens, Sébastien Franceschini, Meike van Leerdam, Haiyu Yang, Sabina Pokharel, Enhong Liu, Puchun Niu, Hanlu Zhang, Saba Noor, Kristof Hermans, Matthieu Salamone, Sumit Sharma

**Affiliations:** 1 Department of Animal Science, College of Agriculture and Life Sciences, Cornell University, Ithaca, NY 14853; 2 Faculty of Bioscience Engineering, Department of Animal Science and Aquatic Ecology, Ghent University, Merelbeke, 9000 Ghent, Belgium; 3 University of Liège, Gembloux Agro-Bio Tech (ULiège-GxABT), 5030 Gembloux, Belgium; 4 Faculty of Veterinary Medicine, Department of Internal Medicine, Reproduction and Population Medicine, Ghent University, 9820 Merelbeke, Ghent, Belgium

## Abstract

The dairy sector should overcome challenges in productivity, sustainability, and data management by adopting intelligent, scalable, and privacy-preserving technological solutions. Adopting data and artificial intelligence (AI) technologies is essential to ensure efficient operations and informed decision making and to keep a competitive market advantage. This paper proposes an integrated, multimodal AI framework to support data-intensive dairy farm operations by leveraging big data principles and advancing them through AI technologies. The proposed architecture incorporates edge computing, autonomous AI agents, and federated learning to enable real-time, privacy-preserving analytics at the farm level and promote knowledge sharing and refinement through research farms and cloud collaboration. Farms collect heterogeneous data, which can be transformed into embeddings for both local inference and cloud analysis. These embeddings form the input of AI agents that support health monitoring, risk prediction, operational optimization, and decision making. Privacy is preserved by sharing only model weights or anonymized data externally. The edge layer handles time-sensitive tasks and communicates with a centralized enterprise cloud hosting global models and distributing updates. A research and development cloud linked to research farms ensures model testing and validation. The entire system is orchestrated by autonomous AI agents that manage data, choose models, and interact with stakeholders, and human oversight ensures safe decisions, as illustrated in the practical use case of mastitis management. This architecture could support data integrity, scalability, and real-time personalization, along with opening up space for partnerships between farms, research institutions, and regulatory bodies to promote secure, cross-sector innovation.

## Highlights

• Presents a multimodal AI ecosystem for dairy farming's big data challenges by 2050.
• Introduces “vulnerability” and “vigilance” dimensions to the big data framework.
• Proposes a layered architecture linking farms, clouds, and research for scalable AI.
• Combines edge computing, federated learning, and autonomous AI agents in an ecosystem.
• Targeted interventions at animal and herd levels, data-driven regional-global decisions.

## Graphical Abstract

**Figure:**
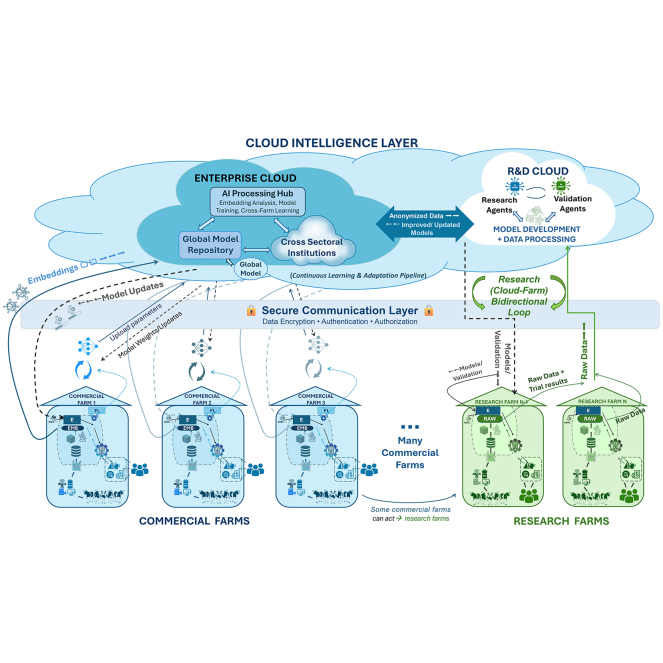
**Summary:** The dairy sector is operating in an environment of rapid technological change, driven by advances in big data and artificial intelligence (AI). To remain competitive and sustainable, farms should adopt intelligent, scalable, and privacy-preserving solutions. This paper presents a comprehensive ecosystem that combines multimodal AI, edge computing, and federated learning to enable real-time, data-driven decision making on farms. With emphasis on privacy, local relevance, and research integration, the framework would enable autonomous AI agents to support everyday farm decisions and long-term planning. The graphical abstract depicts a schematic representation of the proposed framework for a future dairy data ecosystem, illustrating interactions among commercial and research farms, edge devices, and the cloud intelligence layer (enterprise and research and development [R&D] clouds). It shows data and model flows, with solid arrows pointing away from farms and dashed arrows pointing toward farms. E = edge layer; FL = federated learning; RAW = raw data.

Since the early 21st century, the dairy industry has been characterized by increasing integration of big data, artificial intelligence (**AI**), robotics, internet of things (**IoT**), and technologically advanced equipment (Gehlot et al., 2022). Predictive models for precision livestock farming, built on advances in machine learning and deep learning (Cockburn, 2020; Mahmud et al., 2021), range from yield forecasting using historical data (Salamone et al., 2022), behavioral prediction with sensor data (Liseune et al., 2021), health monitoring (van Leerdam et al., 2024), computer vision (Borges Oliveira et al., 2021), and milk mid-infrared spectrometry for herd management (Franceschini et al., 2025). Such applications illustrate how AI-driven tools, which are computational models ranging from simple statistical regressions to advanced machine learning and deep learning methods, can enhance decision-making and optimize production efficiency. However, widespread field applications remain limited, and although the sector generates terabytes of operational data daily, its value is underutilized due to poor integration of monitoring systems (Palma et al., 2025). This challenge ties into the foundational 5 key dimensions of big data; volume, velocity, variety, veracity, and value (Hermans et al., 2018; Nguyen, 2018) which will stay relevant, though their dynamics will evolve. In this evolving landscape, new challenges arise. Two closely connected dimensions that are becoming increasingly important but are rarely or never mentioned in big data discussions and publications are referred to as “vulnerability” and “vigilance.” These emerge as critical additional challenges, highlighting the need for resilient data infrastructure and proactive protection of integrity and privacy. Because AI-driven platforms collect, interpret, and act on large volumes of data transmitted to cloud services or external providers, addressing both vulnerability and vigilance is essential for securing data, ensuring ethical management, and building trust in future-oriented, data-driven systems.

Although many innovative and traditional solutions exist across fields from computer science and engineering to biomedical sciences, the dairy industry has yet to integrate them into a practical data framework. This is why a novel framework is proposed in this paper, combining multiple technologies to provide dairy-specific solutions and maximize data value. The paper is intended for researchers, specialists, farm technology developers, and practitioners engaged with data-driven systems in dairy farming, highlighting opportunities for the application of various concepts rather than detailing a single implementation. It is assumed that readers have basic familiarity with data science; when specialized terms are introduced, concise explanations are provided. First, key concepts of some solutions relatively new to the dairy sector will be described to provide a comprehensive understanding of their potential. These concepts are then integrated into the proposed framework, designed to support the broader dairy ecosystem and set the stage for the rest of the paper.

Dairy farms produce vast, diverse datasets, ranging from milk yield and animal behavior to genomics, feed, environment, soil, and finances, supplemented by external data streams like weather and market data. Their volume and velocity demand scalable storage and near-real-time processing, with integration across sources remaining the major challenge (Cabrera and Fadul-Pacheco, 2021). To address part of these challenges, multimodal AI emerges as a promising solution tackling data heterogeneity. Multimodal AI refers to AI systems designed to process and integrate the input of different data types (e.g., structured data such as sensor readings or tabular information, as well as unstructured data such as text, images, audio, and video) into a single cohesive model (Fuchs, 2023; Arjunan, 2024). This solution captures cross-modal relationships and achieves a more complete, context-aware understanding of input data, surpassing the capabilities of unimodal systems (Ruan, 2024).

As data exceeds local storage, cloud solutions become essential, but sharing sensitive farm information outside the farm raises privacy concerns. Farmers worry that unauthorized access to data (such as yields, finances, or land use) could lead to cyberattacks or misuse, threatening livelihoods and personal privacy (Kaur et al., 2022), a concern that is likely to grow. This is where latent representations captured through dimensionality reduction like embeddings, stored as tensors, can be used. A tensor is a multidimensional array that represents complex data such as images, videos, and time-series (Cichocki, 2018). An embedding is a specific tensor that maps high-dimensional data into a compact, lower-dimensional vector space capturing relationships between data points for efficient storage and processing (Xu, 2021). Typically, data will be first converted into tensors for computation, from which embeddings are learned to create meaningful representations used as input in predictive models (Liseune et al., 2020). Although it is not their primary purpose, embeddings also help to preserve privacy during data sharing by transforming raw sensitive data into an abstract form where the original information can be obscured, making it difficult to reconstruct sensitive details without access to the model that was used to create the embeddings. This allows downstream models to work with the embeddings without ever accessing the raw data, ensuring privacy is maintained. Furthermore, this method is compatible with collaborative research and federated analytics across farms. It is also supported by the concept of federated learning, which is a machine learning approach for decentralized model training, where models train on local data at various sites (e.g., farms) and only aggregated updates are shared with a central server, ensuring privacy and data security without sharing raw data (Dembani et al., 2025).

Another important concept is cloud computing. This paradigm offers an effective solution to train universal global models by integrating data or embeddings from different sources (i.e., farms) and providing the computational power and storage needed for large-scale data analysis (Wang et al., 2024). Through this, farms can offload heavy data processing, model training, and long-term storage to remote data centers, enabling centralized, scalable farm data management. However, cloud computing relies on consistent network connectivity, and for remote farms with unreliable internet, critical alerts such as calving or health warnings may be delayed, jeopardizing time-sensitive interventions. Also, small and medium-sized farms often lack computing power or standardized platforms to manage big data with AI, impeding scalability and adoption of precision approaches (Lokhorst et al., 2019). Edge computing mitigates this by performing storage and computation closer to data sources. This distributed paradigm brings computation and data storage near generation points (e.g., IoT devices or sensors), reducing latency, improving response times, and optimizing bandwidth by processing data locally rather than relying solely on centralized servers (Shi et al., 2016). Edge devices can run lightweight AI models, preprocess data on-site, and limit cloud uploads for tasks such as model training and storage. Additionally, combining edge computing with privacy-preserving embeddings ensures fast, secure processing while protecting sensitive data. Local nodes convert raw sensor inputs into embeddings, perform inference locally, and share only anonymized data with central servers (Žalik and Žalik, 2023). Although edge computing excels in real-time processing, it cannot handle complex tasks such as global model training or long-term storage, which cloud computing enables. Therefore, an integrated edge-cloud framework can be necessary to achieve both real-time performance and scalable, secure data management.

To further enhance on-farm intelligence and reduce manual oversight, autonomous agents can be integrated into this edge-cloud framework. These software entities perceive their environment, make decisions, and act toward specific goals without continuous human intervention. They use techniques such as planning, reinforcement learning, and decision making under uncertainty (Acharya et al., 2025) to optimize outcomes and improve over time by learning from farm data. These agents handle complex, data-driven tasks involving scientific modeling, statistical analysis, and algorithmic computation, which are critical for precision agriculture but fall outside traditional farming skills, and thus typically cannot be performed by farmers. They reduce cognitive load on farmers, researchers, and administrators by translating complex analytics into clear, contextual recommendations tailored to farm size, location, and goals.

To support such AI systems, dedicated research and development (**R&D**) infrastructure is essential. Pilot testing on research farms with advanced instruments for new data generation provides critical feedback to validate models, test technologies, and assess adaptability before wider deployment. These living labs collect high-quality data under real conditions, refining multimodal models, embeddings, edge computing, and data management. Although commercial farms may lack such setups, insights from research farms serve to guide farm agents in developing evidence-based practical solutions by integrating research findings with farm-specific data streams.

By bringing these elements together, the evolving challenges of future dairy farming can be addressed through an approach that incorporates the multitude of V's (volume, velocity, variety, veracity, value, and the newly introduced vulnerability and vigilance). Integrating advanced big data and AI into farm management, a framework is proposed as an intelligent, privacy-preserving system designed to operate within an IoT-enabled, adaptive, and sustainable farm ecosystem, aiming to establish a forward-looking digital dairy ecosystem that enhances resilience, efficiency, and sustainability. The design is conceptualized as a multilayered system across scales, including individual cows, herds, and the industry level, consisting of interconnected components that collect, process, and share data and models, enabling farms to leverage AI while maintaining security and confidentiality. At its core, it combines edge computing, multimodal data, autonomous agentic systems, and a secure communication layer (incorporating data encryption, authentication, and authorization), along with a dedicated R&D environment for continuous performance improvement and an efficient data transfer subsystem (see the graphical abstract). It optionally incorporates federated learning, enabling farms to collaboratively train models while also preserving data privacy and delivering locally optimized outcomes. The overall architecture and data flow between the system components are illustrated in the graphical abstract and the detailed farm view in Figure 1 (refer to https://github.com/Bovi-analytics/Hostens-et-al-2025/blob/main/Model_FrameworFinalFull.jpg for the integrated version).

**Figure 1:**
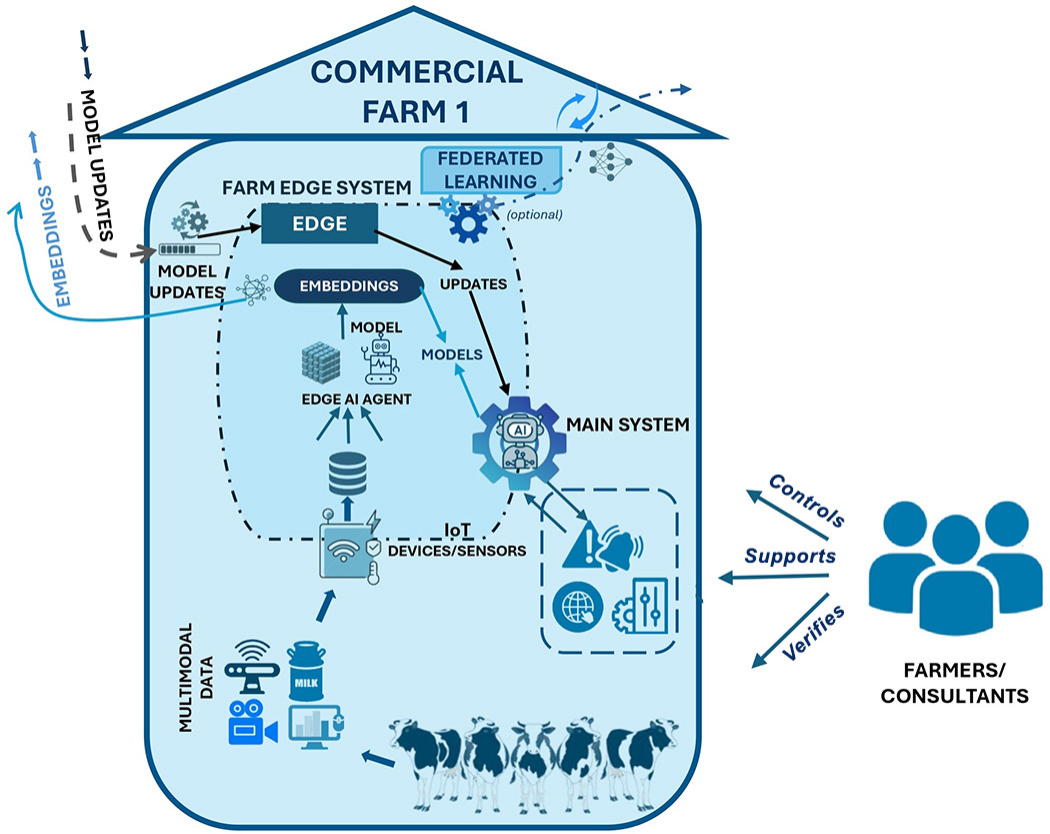
Detailed view of a commercial farm AI system corresponding to the graphical abstract ecosystem. Collected multimodal herd data are processed locally by a farm edge (edge AI agent), which generates embeddings and model updates (optional federated learning) that are transferred to the cloud. The main AI system aggregates updates, issues decisions, and interfaces with farmers and consultants who verify, control, and provide feedback.

The framework contains 2 main components: the farm (commercial and research) and cloud (cloud intelligence layer), with the cloud comprising the enterprise cloud level and the R&D cloud level. At the farm level, multimodal data are collected and processed, including farm management data, sensor data, feed and robot data, and video streams. Here, the edge layer and main system form the foundation for the tasks. These sources gather raw data, which are processed locally using edge computing. Encoders, coordinated by autonomous agents, transform raw inputs into structured numerical forms (tensors) and further into semantic representations (embeddings) that capture relational or contextual meaning (Figure 2). Once generated locally, farm embeddings are securely transmitted to an enterprise cloud environment for further refinement and analysis without exposing any raw farm data. As such, farms could participate in a federated learning network where local edge models train on farm-specific data and only model gradients or weights are shared with the enterprise cloud for aggregation into a global model. This mix of anonymized embeddings for targeted analytics and shared model updates allows farms to benefit from collective intelligence while still preserving privacy. The edge layer supports this architecture by managing local training, ensuring cloud communication, and continuously adapting its output to evolving global insights. It also receives updated models and alerts from the enterprise cloud to optimize operations such as predicting milk production, monitoring animal health, and detecting anomalies or suboptimal conditions. The actual monitoring of farm activities, especially those requiring immediate responses, is primarily carried out by autonomous AI agents running on edge devices, which analyze data in real time, generate embeddings, and trigger alerts or reports. Meanwhile, the enterprise cloud focuses on aggregating data across farms, refining global models and identifying long-term trends and patterns. When necessary, generative AI interface agents can provide adaptive alerts or instructions in local languages or dialects via voice or visual cues, improving decision making and accessibility. Continuous monitoring and response are thus enabled by edge AI agents, whereas cloud-level agents support strategic coordination, model improvement, and global intelligence across the farm network. The system within farms is built to support decisions, with the main system operating under human (farmers and consultants) supervision. The human-in-the-loop setup ensures that everything follows the right protocols, and the final decisions, such as treatments, interventions, or changes on the farm, are made by the farmer or consultant based on the situation and their experience.

**Figure 2:**
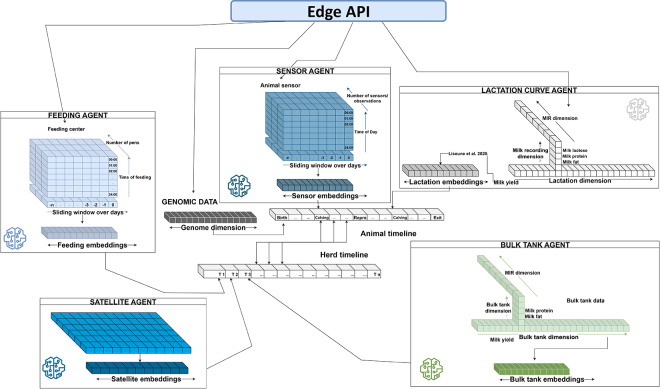
Examples of tensor representations and embeddings in dairy farm data. Multidimensional tensors from milk yield, activity, environment, and genomics are processed locally via standardized application programming interfaces (API) to generate privacy-preserving embeddings. MIR = mid-infrared spectroscopy; Repro = reproduction.

The enterprise cloud level serves as the central hub of intelligence, receiving embeddings from commercial farms and pushing updated models back to ensure continuous adaptation and optimization. Within this layer, several key components interact to support the process, including a global model's repository for storage and distribution, an AI processing hub for embedding analysis, model training, and cross-farm learning, and access for cross-sector institutions for secure knowledge exchange and collaboration. This layer is also directly connected to the R&D cloud level, from which it receives updated models for deployment to farms. In this way, this level integrates diverse embeddings into advanced analytics, connects with institutions for access to global findings (e.g., cross-regional impacts and emerging risks), and acts as a key information source to support R&D decisions, all while ensuring data privacy at every stage. Although autonomous AI agents on edge devices perform real-time monitoring and detect issues such as animal health risks or operational inefficiencies locally to preserve privacy, autonomous cloud agents analyze the resulting embeddings without knowing their farm of origin. These cloud agents can detect broader population-level anomalies and trends, such as disease outbreaks, and generate tailored, data-driven responses. They operate without access to raw data or farm identities, adhering to a private learning paradigm that enables farms to receive personalized insights while keeping their data confidential. If anomalies are found, agentic AI can intervene through a generative AI layer to deliver customized action plans for farmers, industry stakeholders, and policymakers.

Another tailored component within the framework is a targeted connection to research farms, which constitute the R&D cloud, that are tightly integrated into the framework and serve as a testbed for advanced model development and validation. In contrast to commercial farms, research farms could opt to share raw data directly. Researchers as well as autonomous agents in this environment could experiment, refine models, and validate their performance under controlled experimental conditions. This process is critical for generating high-quality operational models (i.e., models used to derive insights and support decision making in farming operations) that will go through the enterprise clouds for dissemination to commercial farms, ensuring that only validated cutting-edge innovations reach the industry. It is well established that benchmarking data and continuous refinement of detection models are crucial to optimizing system performance and relevance in a constantly evolving environment. Although dedicated research farms are assumed to exist, some commercial farms can also share raw data through the cloud for benchmarking and deeper analysis, enabling them to contribute to research efforts and improve embedding generation or discover new insights. These commercial farms may also continue to serve this dual role in the future, meaning they can provide raw data to the research cloud whenever embedding generation needs improvement or new insights are discovered, effectively functioning as research farms when they choose to do so. Such flexibility reflects the evolving landscape where commercial and research farms are not strictly separate entities.

To support ethical scaling and advancement, the enterprise cloud facilitates secure exchange of insights with cross-sector institutions involved in research, innovation, and training. These entities receive only insights derived from embeddings, or, when explicitly approved, the raw data, allowing them to conduct training, research, and collaborative studies without accessing raw sensitive farm data. They contribute their own findings, tools, and updates, feeding emerging research back to the enterprise level. Enterprise AI agents can also be triggered by these insights and trends to initiate actions in the R&D cloud, such as adapting models, suggesting experiments, or flagging risks or opportunities. This arrangement enhances transparency, scientific rigor, and mutual benefit across the ecosystem, driving industry-wide progress in big data and AI applications without extensive data transfer authorization protocols, which are unseen in our industry today.

Sustainability will be embedded into every layer of technological development, including prioritizing energy-efficient machine learning models, edge computing to reduce reliance on centralized data centers, and hosting systems on renewable-powered, green-certified cloud platforms. Data minimization strategies will be applied to reduce storage and energy demands. In addition, acting with greater speed and precision through intelligent decision support systems can help farmers to reduce waste, use resources more efficiently, and lower environmental impacts. Co-developing tools with sustainability experts will ensure that environmental impacts are considered early, and over time, these technologies will inevitably be governed by evolving climate policies and standards.

Maintenance of dairy herd health is a challenge experienced in many dairy farming contexts. In technology-forward farming systems, mastitis monitoring can serve as a practical use case to show how the framework translates data into daily management decisions. Imagine a dairy farm where cows are monitored continuously through sensors, milk meters, and cameras, alongside environmental, health, and farm management data. This information is processed locally via edge computing, transformed into embeddings that represent each cow's physiological and behavioral patterns and the context it lives in, and fed into an on-farm mastitis prediction model. When the model detects a high-risk case, it generates an alert with evidence such as confidence scores, the features driving the decision (e.g., milk conductivity rise, activity drop), and a suggested next step. The farmer or consultant reviews the alert, inspects the cow, and makes the final treatment choice; confirmed case or false alarm provides feedback to the model (called reinforcement learning). Agents supporting this process use generative AI and large language models to deliver farmer-friendly advice in local dialects, for instance by answering the question “Which cows are most at risk for mastitis today, and what should I do?” The agent analyzes farm data, provides individual risk scores, prioritizes cases, and suggests actions based on farm-defined protocols. Importantly, the system is decision-support, not decision-replacement: areas that often involve complex clinical, ethical, or regulatory considerations such as treatment decisions that involve antimicrobial use, reproductive interventions, pain management, culling, or euthanasia remain with humans (farmers, consultants, and veterinarians) and are supported by built-in safeguard mechanisms to ensure responsible and transparent use of AI.

Once a mastitis case is confirmed or rejected, the outcome is used to update the prediction model locally through federated learning. The updated model weights through federated learning and embeddings through edge are sent to the cloud, where enterprise agents track cross-farm or regionally or globally concerning trends (e.g., rising *Escherichia coli* incidence) and can coordinate with veterinary and research partners. In parallel, research and validation agents analyze the pattern using high-resolution data from research farms with gold-standard reference data to validate and refine models, which are redistributed back to production farms, closing the loop between detection and scientific model development. As models are continuously updated, they adapt to factors such as herd size, local disease pressure, climate, and infrastructure, making the framework configurable for diverse production contexts. On large, well-instrumented US farms (e.g., 10,000 cows), the full-stack approach (edge inference, near-real-time embeddings, cloud trend monitoring, and continuous research validation) supports sophisticated, farm-specific tuning. In low-resource settings (e.g., a 100-cow smallholder system in Malawi), the same framework adapts by simplifying components: lower-bandwidth edge models, prioritized low-cost sensors, and emphasis on low-cost interventions. In such contexts, even common smartphones or lightweight local devices can function as edge units, enabling on-farm inference and data handling without reliance on expensive infrastructure.

The proposed framework may face some challenges and limitations. There may be initial reluctance among farmers or farms to share data due to concerns about privacy, misuse, and the potential loss of competitive advantage, which can affect early adoption. Additionally, the computational load and energy demand of large-scale cloud-based analytics raise economic and environmental concerns. Implementing and maintaining such systems also requires technical expertise and skilled personnel, which might not be readily available in all agricultural settings. Ethical considerations are also important; our framework includes human oversight for safety-sensitive decisions such as antibiotic use, but this must be continuously monitored and refined. Despite these challenges, trends in other sectors show that participation tends to grow once the benefits become clear and proper safeguards are in place. Over time, improvements in energy efficiency, the shift to greener cloud infrastructure, and the trend toward smaller, faster, and more affordable devices are likely to reduce many of these barriers. As interfaces become easier to use and automation takes over routine tasks, daily management becomes less demanding. As benefits become clear, farmers and stakeholders can transition to hybrid setups involving periodic cloud synchronization for benchmarking and coordination, eventually moving toward full-stack integration. It is also recognizable that the ideas in the framework can be costly or exclusionary if applied right away. Common barriers such as limited capital, poor connectivity, or lack of technical expertise can be addressed through future cooperative purchasing, extension-led support, vendor-managed deployment models, or public-private and grant-based support for early adoption. Cost-control measures such as data-trust models, subscription or tiered pricing, and shared infrastructure can also help. Over time, ongoing innovation, economies of scale, and improved user accessibility are expected to drive costs down. Although current limitations are real, the direction of technological progress and the evolving mindset within the farming community suggest that practical and sustainable adoption of such data-driven systems is not only possible, but increasingly likely.

In this way, the concept lays a foundation for the future of dairy data operations. Although the future remains inherently dynamic and ever-changing, current trends and rapid advances suggest that such a dairy data ecosystem could guide how farms might operate by 2050. The tools may not develop exactly as described, but the direction is clear: big data and AI can make farming smarter, more efficient, and more sustainable. Integrating technologies such as multimodal AI, edge computing, federated learning, and cloud infrastructure points to a vision of future dairy supported by autonomous AI agents. Over time, these systems could lead to the development of digital twins, which are high-fidelity virtual models that replicate physical farms in near real-time, running predictive models, testing intervention strategies, and supporting long-term planning without disrupting real operations.
